# Mortality after large artery occlusion acute ischemic stroke

**DOI:** 10.1038/s41598-021-89638-x

**Published:** 2021-05-11

**Authors:** Rahul R. Karamchandani, Jeremy B. Rhoten, Dale Strong, Brenda Chang, Andrew W. Asimos

**Affiliations:** 1 Department of Neurology, Neurosciences Institute, Atrium Health, 1000 Blythe Blvd, Charlotte, NC 28203 USA; 2 Information and Analytics Services, Atrium Health, 1000 Blythe Blvd, Charlotte, NC 28203 USA; 3 Department of Emergency Medicine, Neurosciences Institute, Atrium Health, 1000 Blythe Blvd, Charlotte, NC 28203 USA

**Keywords:** Neuroscience, Neurology

## Abstract

Despite randomized trials showing a functional outcome benefit in favor of endovascular therapy (EVT), large artery occlusion acute ischemic stroke is associated with high mortality. We performed a retrospective analysis from a prospectively collected code stroke registry and included patients presenting between November 2016 and April 2019 with internal carotid artery and/or proximal middle cerebral artery occlusions. Ninety-day mortality status from registry follow-up was corroborated with the Social Security Death Index. A multivariable logistic regression model was fitted to determine demographic and clinical characteristics associated with 90-day mortality. Among 764 patients, mortality rate was 26%. Increasing age (per 10 years, OR 1.48, 95% CI 1.25–1.76; *p* < 0.0001), higher presenting NIHSS (per 1 point, OR 1.05, 95% CI 1.01–1.09, *p* = 0.01), and higher discharge modified Rankin Score (per 1 point, OR 4.27, 95% CI 3.25–5.59, *p* < 0.0001) were independently associated with higher odds of mortality. Good revascularization therapy, compared to no EVT, was independently associated with a survival benefit (OR 0.61, 95% CI 0.35–1.00, *p* = 0.048). We identified factors independently associated with mortality in a highly lethal form of stroke which can be used in clinical decision-making, prognostication, and in planning future studies.

## Introduction

Endovascular therapy (EVT) improves functional outcome in selected patients with anterior circulation, large artery occlusion (LAO) acute ischemic stroke (AIS)^1–3^. Randomized trials have demonstrated the benefit of EVT in patients treated within 6 h of Last Known Well (LKW) time^1^. In later time windows, EVT also benefits patients with evidence of salvageable brain tissue on advanced imaging, or a mismatch between clinical exam findings and the volume of infarcted brain tissue^2,3^.

Despite this, LAO AIS remains associated with high mortality. In a meta-analysis of randomized trials demonstrating the benefit of EVT within 6 h, mortality rates in the thrombectomy and standard medical therapy arms were 15% and 19%, respectively^1^. The Endovascular Therapy Following Imaging Evaluation for Ischemic Stroke (DEFUSE 3) trial, which enrolled patients between 6 and 16 h with evidence of salvageable brain tissue, reported mortality rates in the thrombectomy and medical therapy arms of 14% and 26%, respectively^2^. Lastly, the DWI or CTP Assessment with Clinical Mismatch in the Triage of Wake-Up and Late Presenting Strokes Undergoing Neurointervention with Trevo (DAWN) trial, which enrolled patients between 6 and 24 h with a mismatch between exam and imaging findings, showed respective mortality rates of 19% and 18% in the thrombectomy and standard medical therapy groups^3^.

We investigated demographic and clinical characteristics associated with mortality in patients presenting with proximal, anterior circulation LAO AIS. Identifying predictors of mortality in this highly lethal form of stroke has the potential to assist in therapeutic decision-making, prognostication, and for future studies.

## Methods

From November 2016 to April 2019, we conducted a retrospective analysis from a large healthcare system’s prospectively collected code stroke registry of patients with internal carotid artery (ICA) and/or proximal middle cerebral artery (MCA), M1 or M2 occlusions. Ninety-day mortality status from registry follow-up was corroborated with the Social Security Death Index.

Subjects were required to present within 24 h of LKW time. Patients were selected for EVT based on the local guideline for the healthcare system, which closely mirrors recommendations from the American Heart Association/American Stroke Association^4^ (Table 1). Post-thrombectomy reperfusion was determined by neuro-interventionalist calculated Thrombolysis in Cerebral Infarction (TICI) scores. Patients were not required to have been treated with EVT for study inclusion.

**Table 1:** Healthcare system endovascular therapy guideline to determine candidacy for mechanical thrombectomy.

0–6 h	6–16 h	16–24 h
NIHSS ≥ 6	NIHSS ≥ 6	NIHSS ≥ 10
mRS 0–2	mRS 0–2	mRS 0–2
ICA, M1, proximal M2	ICA, M1, proximal M2	ICA, M1, proximal M2
ASPECTS ≥ 6	ASPECTS ≥ 6	ASPECTS ≥ 6
	Core infarct < 70 cc Mismatch volume ≥ 15 cc Mismatch ratio ≥ 1.8	If ≥ 80 yo, core < 21 cc If < 80 yo: NIHSS ≥ 10, core < 31 cc NIHSS ≥ 20, core 31–50 cc

*NIHSS* National Institute of Health Stroke Scale, *mRS* Modified Rankin Scale score, *ICA* Internal carotid artery. *M1* and *M2* Branches of middle cerebral artery. *ASPECTS* Alberta stroke program early computed tomography score.

All patient characteristics were de-identified. Study approval was obtained from the Atrium Health, Carolinas Medical Center Institutional Review Board (IRB, File #07–19-21E). Carolinas Medical Center is a certified Comprehensive Stroke Center. Due to the study design and use of de-identified data, the requirement for informed consent was waived by the IRB.

### Statistical analysis

Descriptive statistics were reported for demographic and clinical characteristics. Baseline clinical characteristics included comorbidities, presenting glucose level, time from LKW to Computed Tomography (CT) imaging, CT image completion, National Institute of Health Stroke Scale (NIHSS), modified Rankin Scale (mRS), thrombolysis in cerebral infarction (TICI) score (good defined as 2b-3), type of treatment (no EVT, EVT only, or IV alteplase and EVT), and revascularization therapy (interaction term for treatment type and baseline TICI score good/poor). Ninety-day mortality was calculated from admission date and confirmed using the Social Security Death Index Masterfile. Univariate analysis was performed to examine risk factors associated with 90-day mortality. A multivariable logistic regression model was fitted to determine demographic and clinical characteristics associated with 90-day mortality. A *p* value of < 0.05 was considered statistically significant.

### Ethical approval

All procedures performed in studies involving human participants were in accordance with the ethical standards of the institutional and/or national research committee and with the 1964 Helsinki declaration and its later amendments or comparable ethical standards. This article does not contain any studies with animals performed by any of the authors.

## Results

Demographic and clinical characteristics are listed in Table 2. In 764 total subjects, mean age was 68 years, 52% were women, and mean NIHSS was 14. Fourteen percent of patients presented with an ICA occlusion, 76% with an MCA occlusion, and 10% had tandem ICA and MCA occlusions.

**Table 2:** Demographic and clinical characteristics (N = 764).

	N	%	Mean	SD
Age (y)	764	–	67.9	15.8
Female	397	52.0	–	–
African-American	210	27.5	–	–
Caucasian	481	63.0	–	–
Asian	12	1.6	–	–
American Indian or Alaskan Native	10	1.3	–	–
Other race	25	3.3	–	–
Hypertension	587	76.8	–	–
Hyperlipidemia	360	47.1	–	–
Diabetes	216	28.3	–	–
Coronary artery disease	17	2.2	–	–
Atrial fibrillation	218	28.5	–	–
Smoking	269	35.2	–	–
Previous ischemic stroke	173	22.6	–	–
Obesity	290	38.0	–	–
Presenting glucose (mg/dL)	–	–	139.7	56.9
Presented in 0–6 h window	550	72.0	–	–
Presented in 6–16 h window	172	22.5	–	–
Presented in 16–24 h window	42	5.5	–	–
CTP completed	592	77.5	–	–
No EVT	369	48.3	–	–
EVT only	202	26.4	–	–
IV TPA and EVT	193	25.3	–	–

*SD* Standard deviation, *EVT* Endovascular therapy, *IV TPA* Intravenous tissue plasminogen activator, *CTP* Computed tomography perfusion.

Seventy-two percent of patients presented in the 0–6 h time window, while approximately 28% presented in the 6–24 h time window. Nearly 40% were treated with IV alteplase. Twenty-five percent were treated with IV alteplase and EVT, and 26% received EVT only.

Mortality rate for the entire cohort was 26% (Table 3). Substantial differences between the survival and mortality groups were seen with respect to age, gender, race, presenting glucose, presence of ICA occlusion, presenting NIHSS, history of hypertension, history of atrial fibrillation, CT perfusion (CTP) core infarction volume (cerebral blood flow, CBF < 30%, IschemaView RAPID), CTP delayed perfusion volume (Time to maximum greater than 6 s, Tmax > 6 s, IschemaView RAPID), treatment with EVT, revascularization therapy, and discharge mRS (Table 3).

**Table 3:** Univariate analysis: 90-day mortality.

	90 day survival				90 day mortality				*p* value
	N	%	Mean	SD	N	%	Mean	SD	
Total patients	568	74.3	–	–	196	25.7	–	–	–
Age (y)	–	–	65.5	15.3	–	–	75.0	14.9	< 0.001
Female	280	49.3	–	–	117	59.7	–	–	0.012
Race									0.004
White	336	59.2	–	–	145	74.0	–	–	
Black	169	29.8	–	–	41	20.9	–	–	
Asian	10	1.8	–	–	2	1.0	–	–	
Other	52	9.1	–	–	8	4.0	–	–	
Obesity	224	39.4	–	–	66	33.7	–	–	0.152
Hypertension	409	72.0	–	–	163	83.2	–	–	0.001
Hyperlipidemia	253	44.5	–	–	96	49.0	–	–	0.282
Diabetes	151	26.6	–	–	56	28.6	–	–	0.555
CAD	14	2.5	–	–	3	1.5	–	–	0.442
Atrial fibrillation	134	23.6	–	–	78	39.8	–	–	< 0.001
Hx ischemic stroke	121	21.3	–	–	48	24.5	–	–	0.354
Glucose (mg/dL)	–	–	136.4	55.7	–	–	149.1	59.2	0.009
NIHSS (median)	–	–	12	–	–	–	20	–	
ICA occlusion	139	24.5	–	–	63	32.1	–	–	0.036
LKW to CS (h)	–	–	4.6	5.3	–	–	4.9	5.5	0.633
CBF < 30%	450	79.2	19.1	33.9	142	72.4	53.4	70.1	< 0.001
Tm > 6 s	450	79.2	112.4	81.7	142	72.4	162.5	109.4	< 0.001
Any EVT	310	54.6	–	–	85	43.4	–	–	0.007
Revascularization therapy									< 0.001
No EVT	258	45.4	–	–	111	56.6	–	–	
EVT poor*	30	5.3	–	–	24	12.2	–	–	
EVT good**	280	49.3	–	–	61	31.1	–	–	
D/C mRS (median)	–	–	3	–	–	–	5	–	< 0.001

*SD* Standard deviation, *Hx* History, *CAD* Coronary artery disease, *NIHSS* National Institute of Health Stroke Scale, *ICA* Internal carotid artery, *LKW* Last known well time, *CS* Code stroke, *CBF* Cerebral blood flow, *Tm* > *6 s* Time to maximum greater than 6 s, *EVT* Endovascular therapy, *D/C* Discharge, *mRS* Modified Rankin scale score. Percentages listed are with respect to subjects in individual ‘90 day survival’ or ‘90 day mortality’ groups, except for Total Patients.

*EVT poor = EVT with thrombolysis in cerebral infarction score 0–2a.

**EVT good = EVT with thrombolysis in cerebral infarction score 2b–3.

In the multivariable model (Table 4), increasing age (per 10 years, OR 1.48, 95% CI 1.25–1.76; *p* < 0.0001), higher presenting NIHSS (per 1 point, OR 1.05, 95% CI 1.01–1.09, *p* = 0.01), and higher discharge mRS (per 1 point, OR 4.27, 95% CI 3.25–5.59, *p* < 0.0001) were associated with higher odds of mortality. Good revascularization therapy (TICI 2b-3), compared to no EVT, was associated with a survival benefit at 3 months (OR 0.61, 95% CI 0.35–1.00, *p* = 0.048).

**Table 4:** Multivariable logistic regression analysis for 90-day mortality.

	Unit	OR	Lower CI	Upper CI	*p* value
Age	10 years	1.48	1.25	1.76	< 0.0001
Female versus male	–	0.84	0.50	1.40	0.503
mRS at discharge	1 point	4.27	3.25	5.59	< 0.0001
NIHSS	1 point	1.05	1.01	1.09	0.010
EVT poor versus no EVT	–	1.34	0.58	3.10	0.501
EVT good versus no EVT	–	0.59	0.35	1.00	0.048

*OR* Odds ratio, *CI* Confidence interval (Wald), *mRS* Modified Rankin Scale score, *NIHSS* National Institute of Health Stroke Scale, *EVT poor* EVT with thrombolysis in cerebral infarction score 0–2a, *EVT good* EVT with thrombolysis in cerebral infarction score 2b–3.

## Discussion

Our retrospective analysis from prospectively collected data on 764 subjects showed an independent association between age, NIHSS, and discharge mRS with 90-day mortality in patients with ICA and/or proximal MCA occlusions. Good revascularization therapy, compared to no EVT, improved 90-day survival.

Numbers-needed to treat for functional independence in the Highly Effective Reperfusion evaluated in Multiple Endovascular Stroke (HERMES) trial, DEFUSE 3, and DAWN cohorts were 2.6, 2, and 2.8, respectively^1–3^. While a meta-analysis suggests that EVT improves 3-month survival compared to best medical therapy^5^, EVT has demonstrated a statistically significant mortality benefit in only a single randomized trial^6^. Identifying subsets of patients at higher risk of death is thus especially important, as this can guide clinical management, assist family discussions and prognostication, and help plan future clinical trials. Modifiable risk factors may be targeted in future studies, additional relevant clinical endpoints for studies can be elucidated, and evidence-based therapies for improvement in functional outcome, such as EVT, may be refined in order to reduce mortality risk.

Other groups have identified factors associated with mortality at various time points after stroke, including in-hospital models and 1-year predictive models^7–22^. However, exclusive to LAO patients, these models have been specifically designed and/or validated to predict either good or poor functional outcome^17–22^. Our work is unique in targeting LAO patients that have been treated with best medical therapy + /− EVT, with the dependent variable of mortality at 90 days, rather than poor outcome (often defined as mRS 4–6).

Among the common mortality associations in other studies include age^7–10,14,16^, initial stroke severity^7–9,13–16^ presenting glucose^8,16^, pre-morbid function^7–9^, and history of atrial fibrillation^8–10,15^. We similarly found that age, initial stroke severity, presenting glucose, and history of atrial fibrillation were significant univariate associations with mortality risk, though among these factors, the multivariable model demonstrated independent mortality associations with increasing age, presenting NIHSS, and discharge mRS. These differences may be explained by the heterogeneity of the patient cohorts and treatment algorithms.

While age^17–22^ and stroke severity^17–21^ have previously been incorporated into functional outcome prediction models for LAO patients, as well as into prognostic models for non-selected ischemic stroke patients^7–10,15^, our findings of an independent association with mortality in strictly the LAO population is novel. In addition, to our knowledge, discharge functional outcome has not previously been demonstrated to predict mortality, though we have recently shown its importance in predicting 90-day outcome in basilar artery occlusion patients treated with EVT^23^.

Moreover, while good revascularization may improve survival in the thrombectomy population^24–26^, our study demonstrated that TICI 2b-3 revascularization, compared to no EVT, produced a survival benefit. This may seem intuitive, as patients receiving no EVT may be categorized into the “no revascularization” or “poor revascularization” group of a thrombectomy-only study. However, we believe this is still significant in that this was shown in a cohort of patients that included exclusively medically managed LAO patients, indicating that the treatment effect of only medical therapy did not modify this benefit. Future studies may build on our findings to develop mortality prediction models specifically in this subset of patients, as other groups have done for functional outcome^17–22^.

The logistic regression model showed that each increasing decade of life elevated odds of 90-day mortality by nearly 1.5 times. While age is a non-modifiable risk factor, quantification of its contribution to mortality risk is helpful during hospital management and prognostication. It should be emphasized that thrombectomy should still be offered to older patients, as the clinical benefit in favor of treatment for functional improvement has been demonstrated across the spectrum of age ranges in randomized trials^1–3^. Subgroup analyses from early and late-window thrombectomy trials demonstrate a trend for higher likelihood of functional independence in older patients than younger patients treated with EVT, compared to similar age patients treated with best medical therapy^1–3^. Further studies may focus specifically on the impact of age on risk of mortality in this population.

One might also hypothesize that discharge mRS impacts risk of 90-day mortality. In our study, an over fourfold odds of death were found with each increasing point on the mRS at discharge. Discharge mRS may be impacted by multiple factors, including pre-morbid mRS, presenting NIHSS, treatment with EVT, and medical co-morbidities, all of which were investigated in our study. Another factor that may influence discharge mRS is the institution of early rehabilitation. Trials conducted thus far instituting very early rehabilitation after stroke have shown mixed results, though initiation of rehabilitation within 2 weeks appears to be beneficial^27^. Targeting specific therapies with more defined time windows may be a focus of future studies impacting post-stroke mortality.

Elevated presenting NIHSS was associated with increased mortality risk in our study, and while presenting NIHSS may not be able to be specifically impacted, it can be used in risk assessments for prognosis. In addition, targeting primary and secondary prevention may reduce not only risk of incident stroke, but may impact collateral blood flow status^28^, and thereby presenting stroke severity. This specific clinical question requires additional study.

Good reperfusion, defined as achieving TICI 2b-3, has been associated with improved functional outcome after LAO stroke^29,30^ and may improve survival in the subset of LAO patients that are treated with EVT^24–26^. Our study demonstrated that compared to no EVT, good revascularization improved odds of survival. This further emphasizes the importance of achieving good reperfusion with EVT, and is an example of reinforcing an existing, evidence-based treatment for functional improvement for a potential survival benefit. TICI 2b-3 revascularization was achieved in 86% of patients receiving EVT in our study, and in 76% and 84% of patients in DEFUSE 3 and DAWN, respectively^2, 3^. In HERMES, TICI 2b-3 revascularization was achieved in a low of 59% of patients in MR CLEAN^31^ up to a high of 88% of subjects in SWIFT-PRIME^32^.

The mortality rate for our entire cohort was 26%, higher than that reported in the medical and EVT arms of prior thrombectomy trials^1–3,5^. However, elevated in-hospital mortality has been reported for EVT patients treated outside of clinical trials^33^. In addition, subjects who met inclusion criteria for DEFUSE 3 and DAWN were required to have a mismatch between infarcted brain tissue and salvageable brain tissue, or a mismatch between infarcted tissue and clinical exam findings. As such, most patients in DEFUSE 3 or DAWN likely had “slow-growing” infarcts by meeting inclusion criteria for the trials. Accordingly, subjects randomized to the medical therapy arms of the trials might be expected to have better outcomes than subjects who did not meet trial criteria. An extension of this so-called “late-window paradox”^34^ for good outcome may also apply to mortality, such that meeting trial enrollment criteria may offer a protective survival benefit. In our study, late window (6–24 h) patients comprised 28% of all subjects and just over 50% of all subjects were treated with EVT.

Moreover, the elevated number of M2 branch occlusions in our study (over 30% of total subjects, compared to 8 total patients in DEFUSE 3 and DAWN and 8% in HERMES), higher ischemic core volume (mean of 27 cc, compared to 10 cc in DEFUSE 3 and 8 cc in DAWN), and inclusion of subjects with mRS 0–2 (rather than 0–1) for EVT may have contributed to our higher mortality as well.

Our study had several limitations. Pre-morbid mRS scores were not consistently available and could not be analyzed, though discharge mRS scores were captured. While obtained in over three-quarters of patients, CTP was not routinely performed on every LAO patient, limiting the conclusions that may be drawn from these data. The focus of our study was on mortality, though some might consider an mRS score of 5 to be an equally bad or worse outcome than death. The sub-category of patients with mRS 5 was not quantified at 3 months, as outcome data on patients not treated with alteplase or EVT was not routinely captured at 90 days. Some patients died as a direct result of stroke, while others died after withdrawal of care when meaningful neurological recovery appeared exceedingly unlikely. Patients who died as a result of withdrawal of care were not independently analyzed.

## Conclusion

Increasing age, higher presenting NIHSS, and worse discharge functional outcome were associated with 90-day mortality after LAO AIS in our study. Good revascularization after EVT, compared to no EVT, improved survival. These factors may be used in clinical settings to assist in treatment, prognostication, resource allocation, financial planning, and in future trials.

## Data Availability

The datasets generated during and/or analyzed during the current study are available from the corresponding author on reasonable request.
